# Micro-planning at scale with key populations in Kenya: Optimising peer educator ratios for programme outreach and HIV/STI service utilisation

**DOI:** 10.1371/journal.pone.0205056

**Published:** 2018-11-01

**Authors:** Parinita Bhattacharjee, Helgar Musyoki, Ravi Prakash, Serah Malaba, Gina Dallabetta, Tisha Wheeler, Stephen Moses, Shajy Isac, Richard Steen

**Affiliations:** 1 Centre for Global Public Health, University of Manitoba, Winnipeg, Canada; 2 National AIDS and STI Control Programme, Ministry of Health, Nairobi, Kenya; 3 Karnataka Health Promotion Trust, Bangalore, India; 4 Partners for Health and Development in Africa, Nairobi, Kenya; 5 Bill & Melinda Gates Foundation, Washington DC, United States of America; 6 Office of HIV/AIDS, Bureau for Global Health, U.S. Agency for International Development, Washington DC, United States of America; 7 Department of Public Health, Erasmus MC, University Medical Center Rotterdam, Rotterdam, The Netherlands; Purdue University, UNITED STATES

## Abstract

Peer education with micro-planning has been integral to scaling up key population (KP) HIV/STI programmes in Kenya since 2013. Micro-planning reinforces community cohesion within peer networks and standardizes programme inputs, processes and targets for outreach, including peer educator (PE) workloads. We assessed programme performance for outreach–in relation to the mean number of KPs for which one PE is responsible (KP:PE ratio)–and effects on HIV/STI service utilisation. Quarterly programmatic monitoring data were analysed from October 2013 to September 2016 from implementing partners working with female sex workers (FSWs) and men who have sex with men (MSM) across the country. All implementing partners are expected to follow national guidelines and receive micro-planning training for PEs with support from a Technical Support Unit for KP programmes. We examined correlations between KP:PE ratios and regular outreach contacts, condom distribution, risk reduction counselling, STI screening, HIV testing and violence reporting by KPs. Kenya conducted population size estimates (PSEs) of KPs in 2012. From 2013 to 2016, KP programmes were scaled up to reach 85% of FSWs (PSE 133,675) and 90% of MSM (PSE 18,460). Overall, mean KP:PE ratios decreased from 147 to 91 for FSWs, and from 79 to 58 for MSM. Lower KP:PE ratios, up to 90:1 for FSW and 60:1 for MSM, were significantly associated with more regular outreach contacts (p<0.001), as well as more frequent risk reduction counselling (p<0.001), STI screening (p<0.001) and HIV testing (p<0.001). Condom distribution and reporting of violence by KPs did not differ significantly between the two groups over all time periods. Micro-planning with adequate KP:PE ratios is an effective approach to scaling up HIV prevention programmes among KPs, resulting in high levels of programme uptake and service utilisation.

## Introduction

Kenya was affected early and severely when the human immunodeficiency virus (HIV) epidemic erupted in eastern Africa in the 1980s. Low condom use in sex work and high rates of ulcerative sexually transmitted infections (STIs) combined to drive extremely rapid HIV transmission rates. [1,2] HIV prevalence among female sex workers (FSWs) surpassed 80% by the late 1980s, and more than 40% of uncircumcised men with genital ulcers seroconverted to HIV after a single sex work exposure. [2] Yet, effective programmes succeeded in raising nearly non-existent condom use among Nairobi sex workers to 80% of reported sexual contacts within a few years. [1,3] STI rates fell abruptly and HIV prevalence progressively declined in Nairobi during the 1990s. [3,4] HIV continued to decline nationally as FSW programmes were extended to other towns. However, programme focus then shifted to the general population, in Kenya and the region, in part because research underestimated the contribution of sex work to overall HIV transmission in mature HIV epidemics. [5,6]

Attention returned to FSWs, along with men who have sex with men (MSM) and people who inject drugs (PWID), in 2009, when a modes of transmission study in Kenya estimated that at least one-third of all new HIV infections could be attributed to these three key populations (KPs). [7,8] Kenya conducted a mapping exercise of key populations in 2012, and through a consensus meeting estimated that the country had approximately 133,675 FSWs, 18,460 MSM and 18,327 PWID. [9] Subsequently, HIV prevention programs for KPs were scaled up in 31 out of 47 counties with 81 implementation partners, funded by U.S. President's Emergency Plan for AIDS Relief (PEPFAR) and the Global Fund for AIDS, Tuberculosis and Malaria (GFATM). The KP Programme in Kenya, led by the National AIDS and STI Control Programme (NASCOP) and the National AIDS Control Council (NACC), adopted a combination prevention approach with a balanced focus on behavioural, biomedical and structural interventions. [10] NACC and NASCOP, with national and international partners, including KP-led organisations, have developed policies and guidelines to define and guide KP programme implementation. [10,11] NASCOP, through its Technical Support Unit funded by the Bill & Melinda Gates Foundation (BMGF) and implemented by the University of Manitoba, provides training, supportive supervision and mentoring to the implementing partners to ensure effective implementation of the national KP programme. [10,12]

It was recognised that HIV prevention and care programmes with KPs are different in important ways from those designed for the general population; the latter aim to test as many people as possible at least once (‘know your status’), link them to anti-retroviral therapy (ART) and support high retention to suppress viral load. [13] Testing may or may not be guided by risk assessment or include follow-up programming for those testing HIV-negative. Programmes with KPs, on the other hand, require sustained, high-intensity interventions to address behavioural, biomedical and structural factors, tailored to local context and led by KP communities. [14] Regular and sustained contact with KPs, irrespective of their serostatus, is important to support those who are HIV-negative to remain negative, while maximising retention for HIV-positives. [15–20]

In line with global guidance, peer-to-peer outreach and education have been defined as key strategies of the Kenya KP programme. [10,16,17] National guidelines mandate KP programmes to adopt a community-led outreach strategy, involving peer educators (PEs) and outreach workers, and set standards–ratios of KPs to peer educators (KP:PE ratio), criteria for selecting PEs, etc. Peer educators are active KPs who do outreach at sites where they practice sex work or seek sex partners. They should be knowledgeable about the local context, acceptable and accountable to the community and programme, able to maintain confidentiality, and have good listening, communication, and interpersonal skills. PEs also commit to being available to KPs in case of problems such as violence [16,17].

In Kenya, PEs use micro-planning to organise their work. Micro planning is an approach that aims at efficient scale-up of service delivery by identifying and prioritising key locations, hotspots, networks and individuals with the greatest need of services. Micro-planning decentralises outreach planning, management and monitoring, facilitates engagement of KPs by the peer educator, and empowers the PE to make decisions on how to best reach KPs and address their needs [21–23]. The approach employs a set of simple tools–including site mapping and analysis, peer calendar, opportunity gap analysis–which allow PEs and outreach workers to collect and use data in their work with KPs regardless of HIV status. This information is updated regularly to guide outreach activities.

Micro-planning helps peer educators transition from being passive data gatherers to active site managers who analyse data from their spots and networks. Such information is used by PEs to follow up with KPs they are responsible for, understand their risks and vulnerabilities and plan outreach and services based on their needs. [22]. Micro-planning has been used extensively in Kenya and in India [21–24]. With support from BMGF, PEPFAR and other donors, micro-planning methods are being adapted and applied elsewhere [25].

While the literature supports the effectiveness of peer education, there is less evidence on operational aspects of micro-planning, or its impact on service uptake, and on behavioural, biological and structural outcomes. Some studies have shown benefit from higher frequency PE contact with FSWs [26–28], but evidence to inform budgeting and practical outreach planning and staffing, such as KP:PE ratios, is lacking. This study aims to: 1) assess optimal KP:PE ratios for effective outreach with micro-planning; and 2) clarify the relationship between the KP:PE ratio and key programme outcomes, including regular outreach contact, condom distribution, risk reduction counselling, STI screening, HIV testing, and violence reporting by KPs.

### Programme description

**Fig 1:**
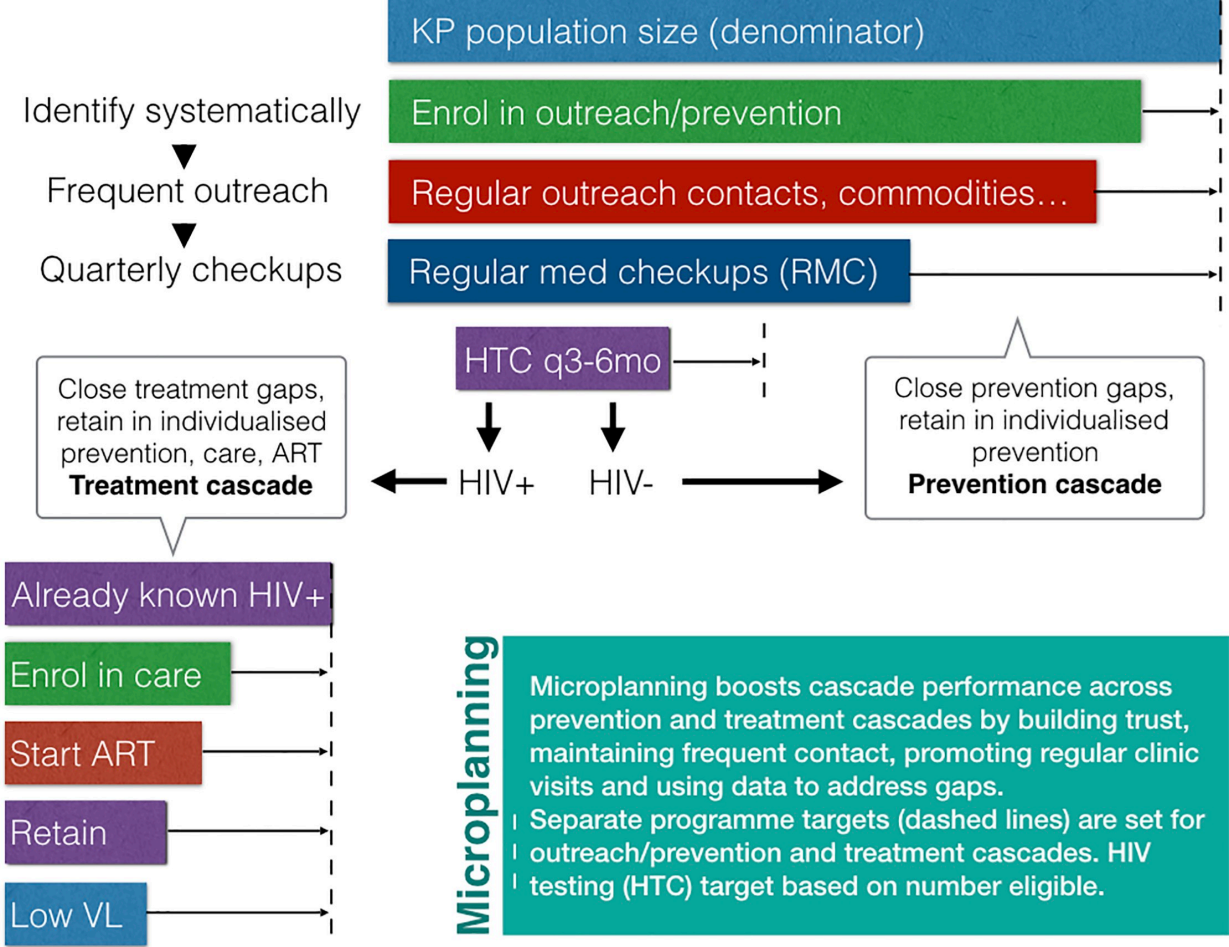
Micro-planning boosted cascade model for key populations. Abbreviations: KP: Key Populations, RMC: Regular Medical Checkup, HTC: HIV Testing and Counselling, ART: Anti-retroviral Therapy, VL: Viral load.

Peer-led outreach, a core component of KP programmes in Kenya, is both a behavioural change intervention, and a means to link KPs to clinical services and structural interventions. PEs work part-time as incentivized volunteers and are expected to conduct regular outreach (providing risk-reduction information, skills, and commodities), through one-to-one contacts with KPs in hotspots where the PE normally operates. Micro-planning provides a structured approach to outreach work and sets operational standards (Fig 1). The expected workload of each PE, or KP:PE ratio, varies in Kenya for different KP subgroups, locations and implementing partners. National guidelines include *ad hoc* ranges– 60–80 FSWs per PE and, due to the more dispersed nature of the MSM community, 30–40 MSM per PE. If a hotspot or cluster of hotspots has more than 80 FSWs or 40 MSM, the programme is expected to recruit a second PE to maintain KP:PE ratios. The Kenya national guidelines mandates programmes to pay an honorarium of Ksh 3500 (35 USD) per month to every peer educator [10].

PEs are expected to conduct regular outreach with the KPs they are responsible for. While the expectation is for at least monthly contacts, high-risk situations often require more frequent contacts, while high KP mobility may reduce the frequency. During outreach contacts, PEs provide information, build trust in the programme, and supply condoms and lubricants according to need. PEs also promote preventive and curative services and mobilise KPs to visit the programme clinic quarterly for routine checkups (Fig 1). These include risk reduction counselling, STI screening, and HIV testing. Support to report and address any incidents of violence is provided as and when needed. To deliver these services effectively, PEs need to be familiar with the “hotspots” where sexual contact is commonly made, and to understand the needs of KPs in those spots. Micro-planning provides PEs with skills to determine how to reach the KPs they are responsible for, assess the needs of the KPs, and address these needs effectively. Simple tools for collecting and using data empower PEs to monitor their work at local hotspot level, find solutions to challenges and contribute to overall programme efficiency [10,21,22].

## Methodology

### Data description

We used routine programme monitoring data for the period October 2013 to September 2016 to describe the scale-up of KP programmes in Kenya. We then used a subset of these data, as detailed below, to assess changes in the key population to peer educator (KP:PE) ratio and its relationship with key outcomes related to use of HIV/STI programmes and services. Each implementing partner monitors the uptake and utilisation of clinical and non-clinical services. These individual-level data on outreach, service uptake, and violence reporting are aggregated at implementing partner and county-level and reported to NASCOP on a quarterly basis in a standard format. These data are collected using paper-based registers/formats and are updated continuously by data entry operators hired by implementing partners. Monitoring and evaluation officers from the NASCOP Technical Support Unit provide support and conduct quality checks. Aggregate data submitted quarterly by implementing partners to NASCOP were used for the present analysis.

We performed data cleaning and smoothing prior to data analysis. In the course of programme scale-up from 2013–16, new counties and implementing partners were added and dropped due to reorganisation and reprioritisation of donor funding. In some counties, new implementing partners were added/replaced, or targets of existing partners were revised, resulting in inconsistent data. To overcome these inconsistencies, data smoothing was done by excluding data from counties lacking reliable data for all three years (12 quarters). We used mapping data from 2012 for estimates of FSWs and MSM in the counties [29]. No new size estimates have been conducted in the country since 2012 so the national programme uses the county estimates derived from the 2012 mapping and national estimates derived from the 2013 mapping consensus report. Table 1 summarises the data used for the KP:PE ratio sub-analysis. Data from 22 FSW counties and 18 MSM counties with consistent data represent about 90% of the total estimated KPs in the country.

**Table 1:** Profile of the sample included in KP:PE ratio analysis.

Indicators	Female Sex Workers			Men who have sex with men		
	2013–14	2014–15	2015–16	2013–14	2014–15	2015–16
Total number of reporting counties	30	31	29	23	22	24
Number of counties with consistent reporting over three years	22	22	22	18	18	18
% total estimated population covered in counties with consistent reporting*	89.8	88.1	91.0	95.4	98.1	92.9

* using 2012 population size estimates

### Outcomes and measures of interest

The relationship between strong peer programme inputs–numbers of PEs trained and supported in micro-planning–and regular outreach contacts is the primary outcome of interest, as peer outreach has been shown to be a critical ‘gateway’ to other services. [27] The analysis also includes several secondary ‘service utilisation’ outcomes, which may in turn be catalysed by frequent outreach contacts. The operational strength of peer micro-planning efforts was defined for this analysis as the mean number of KPs for which one PE is responsible (KP:PE ratio). The measure of KP:PE ratio was derived from the total number of KPs to be covered (donor target) divided by the number of active PEs reported by the programme. Outcome indicators such as the proportion of KPs regularly contacted through outreach, counselled on risk reduction, screened for STIs, tested for HIV, condom distribution, and who reported violence, were calculated using donor targets as denominators. It should be noted that these measures likely underestimate performance for some indicators. The target for HIV testing, for example, is the same as the ‘reach’ target, which includes known HIV-positive KPs who are not eligible for HIV testing. Condom distribution does not take into account fluctuating condom requirements, and violence reporting does not factor in reductions in violent incidents (which is a separate aim of the programme).

The variable for ‘regular outreach contact’ comes from quarterly data and indicates the number of individual KPs contacted (at least once) during each quarter. Although monthly PE contacts are recommended by the Kenya KP programme, individual-level data on monthly contacts were not available for analysis. The condom distribution indicator was calculated using the total number of condoms distributed during the quarter (through direct distribution by the peer educators and indirect through venues and condom depots that the peer educators fill regularly) divided by the number of KPs contacted during the same quarter.

### Analysis

Bivariate analysis in the form of percentages and means was used. All the percentages and means were calculated for each quarter and then averaged to arrive at an estimate for the year. Along with the mean KP:PE ratio, the inter-quartile range (IQR: 25th and 75th percentiles) was calculated. We also grouped the counties into two clusters, above or below/equal to the average KP:PE ratio, and compared primary and secondary outcomes for those clusters. A two-sample proportion test using Z-statistics was performed to detect the statistical significance of the difference in service uptake between the two clusters. All statistical analyses were performed using STATA software (version 14.0).

### Ethical considerations

All data were collected through routine programme monitoring by implementing partners and sent to NASCOP anonymously without any identifiers. Ethical approval was received from the Kenyatta National Hospital-University of Nairobi Ethical Review Committee, approval number P647/11/2017, to conduct secondary data analysis of the monitoring data.

## Results

### Scale-up and overall programme performance

**Fig 2:**
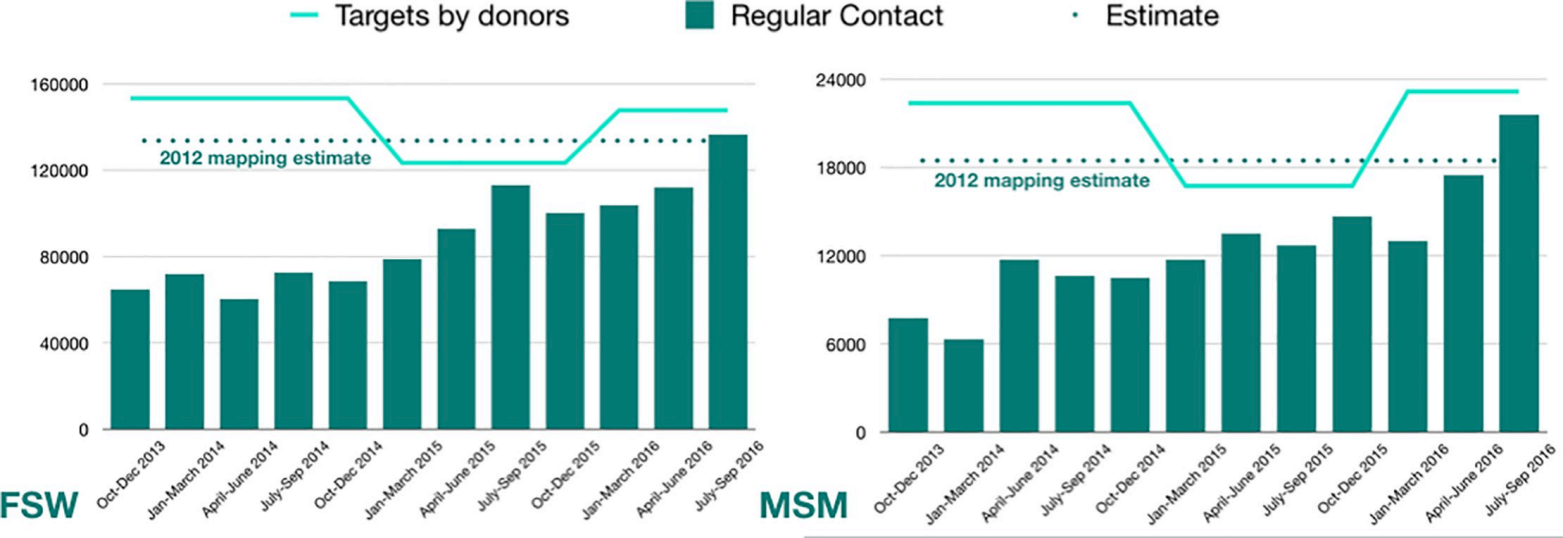
Quarterly outreach contacts, population size estimates and donor targets (31 FSW counties and 24 MSM counties). Abbreviations: FSW: Female sex workers, MSM: men who have sex with men [30].

Key population size estimates for Kenya in 2012 were 133,675 FSW and 18,460 MSM. From 2013 to 2016, KP programmes were scaled up rapidly, reaching approximately half the estimated KPs in the first year. Micro-planning was introduced from the beginning as the standard approach for peer-led outreach. Although donor targets for implementing partners fluctuated over the period, quarterly reach (unique individuals reached at least once during the quarter) averaged 85% for FSWs and 90% for MSM over the year 2015–16 (Fig 2) [30].

### Peer ratios and outreach

Since maintaining strong outreach is a primary aim of the Kenya KP programme, regular outreach contact is a priority programme outcome. Implementing partners hired PEs and outreach workers to conduct outreach and KP registration in sites where FSW and MSM solicit for sex or cruise.

**Fig 3:**
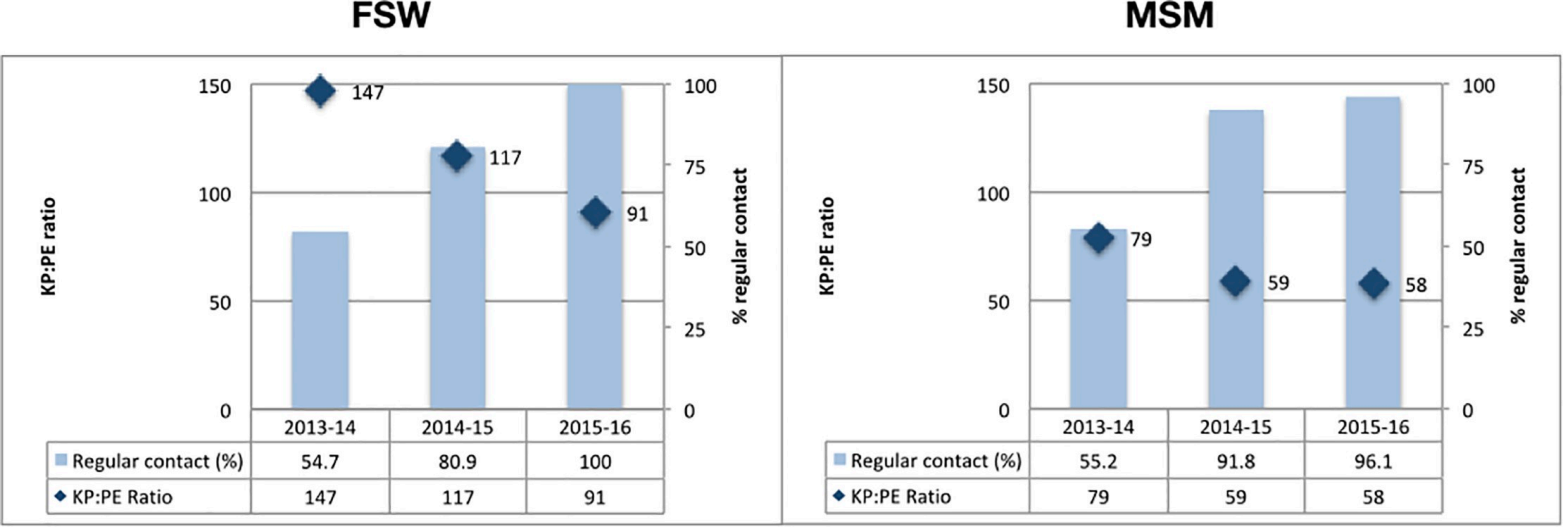
Regular outreach contacts and PE:KP ratio (22 FSW counties and 18 MSM counties). Abbreviations: FSW: Female sex workers, MSM: Men who have sex with men, KP: Key Populations, PE: Peer educator [30].

The KP:PE ratio reflects an important input that has varied over time and by implementing partner (Fig 3). Data from a subset of consistently reporting counties (22 for FSW and 18 for MSM, as described in Methods), shows that the KP:PE ratio declined over the years, while average regular outreach contacts increased. In 2013–14, with a high KP:PE ratio of 147:1, regular contacts averaged only 55% of FSWs each quarter. By 2015–16 with a KP:PE ratio of 91:1, 100% of the FSWs against the donor target were receiving regular contacts at least quarterly. Similarly, for MSM, in 2013–15, with a KP:PE ratio of 79:1, only 55% of the MSM population against the donor target were being reached regularly. By 2015–16 when the KP:PE ratio was 58:1, 96% of MSM were receiving regular contacts.

This time trend suggests that lower KP:PE ratios increase regular outreach contacts among FSWs and MSM. The findings also appear to confirm that MSM programmes may need lower KP:PE ratios than FSWs, at least in the Kenya context, to maintain regular outreach coverage.

### Peer ratios and other service outcomes

**Fig 4:**
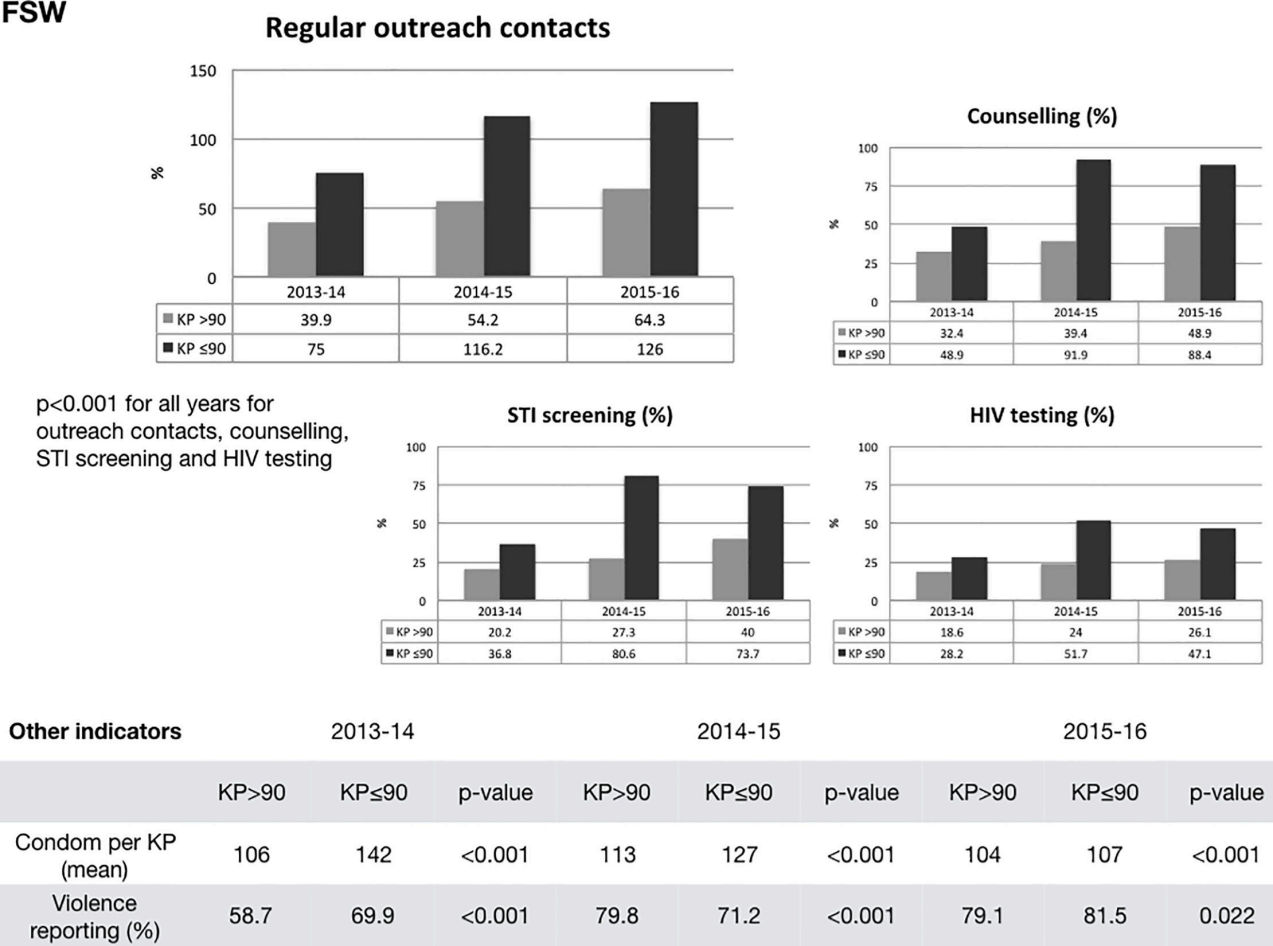
Regular outreach contacts and service utilisation by PE:KP ratio (22 FSW counties). Abbreviations: KP: Key Populations, PE: Peer educator, STI: Sexually transmitted infections, HIV: Human immune deficiency virus [30].

We also looked at peer ratios for evidence of an effect on other important KP service outcomes. Using the same sub-sample of consistently reporting counties, we stratified the FSW groups into two clusters, one with an FSW: PE ratio of 90:1 or lower, the other higher than 90:1. Fig 4 shows that in all three years, the clusters with a KP:PE ratio of 90:1 or lower reported significantly higher regular outreach contacts (p<0.001), higher uptake of risk reduction counselling (p<0.001), higher STI screening in clinics (p<0.001) and higher HIV testing (p<0.001). These findings were somewhat less consistent for condom distribution and violence reporting.

**Fig 5:**
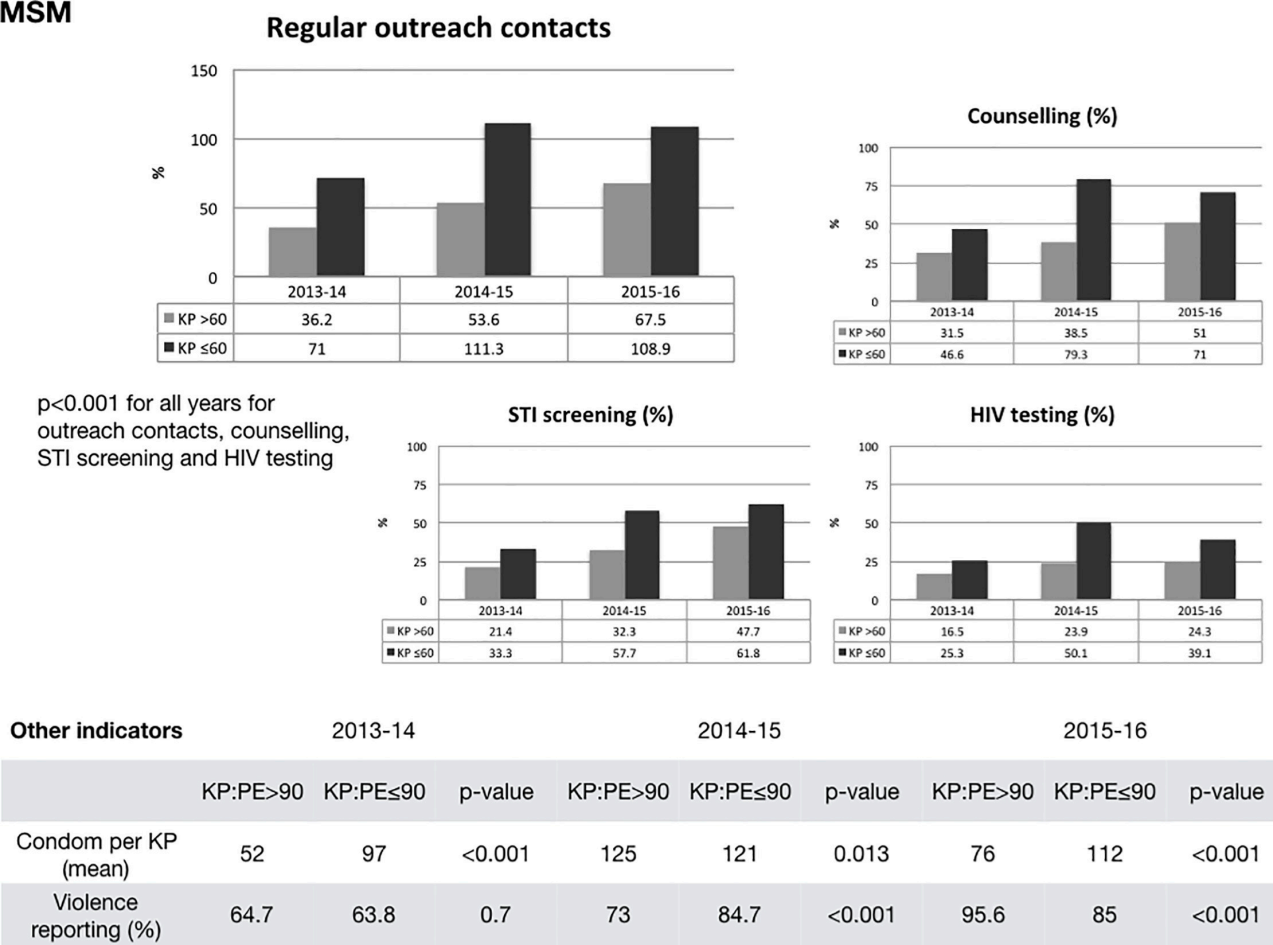
Regular outreach contacts and service utilisation by PE:KP ratio (18 MSM counties). Abbreviations: KP: Key Populations, PE: Peer educator, STI: Sexually transmitted infections, HIV: Human immune deficiency virus [30].

Similarly, we stratified the MSM programmes across consistently reporting counties into clusters with a KP:PE ratio of 60:1 or lower, and of higher than 60:1. Fig 5 shows that the lower KP:PE cluster reported significantly higher regular contacts with MSM (p<0.001), higher uptake of risk reduction counselling (p<0.001), higher STI screening in clinics (p<0.001), and higher HIV testing (p<0.001). As with the FSW clusters, the findings were less consistent for condom distribution and violence reporting.

For both FSWs and MSM, KP:PE ratios below the observed thresholds not only significantly improved outcomes such as outreach contacts, for which PEs have direct responsibility, but also regular clinic attendance for a range of complementary HIV/STI prevention, screening and treatment services.

## Discussion

Well-implemented interventions for HIV/STI prevention, care and treatment with key populations slow HIV/STI transmission and extend treatment to many in need. [31,32] The KP programme in Kenya, scaled up rapidly over three years, is reaching an estimated 85% of FSWs and 90% of MSM across the country. The scale-up, targeting and intensity of those interventions is guided and managed using micro-planning methods supported by a dedicated technical support unit. What are the main outcomes of value to KP communities, and how do related programme inputs contribute to these?

The Kenya KP programme has adopted a combination prevention approach, requiring implementing partners and donors to offer a standardised set of behavioural, biomedical and structural interventions. Emphasis is placed on prevention skills, use of clinical services, and preventing violence. Performance targets are set by the national programme and donors, for a range of priority programme indicators of coverage, process, and outcomes. Micro-planning is used as a standard approach to strengthen outreach for all implementing partners. A Technical Support Unit provides training and mentoring support to all the implementing partners to ensure that the programme is of high quality and adheres to the national guidelines. After three years of systematic implementation, national-level programme data show, in addition to high levels of scale and coverage, significant increases in regular outreach to both FSWs and MSM. These more regular contacts in turn have resulted in more frequent use of clinic services, including HIV counselling, STI check-ups, and HIV testing.

Available routine monitoring data did not permit extending the above analysis of peer ratios to other areas of service delivery, such as antiretroviral treatment (ART) linkage and retention. However, population-based annual survey data conducted by NASCOP show high linkage to care and ART for KP identified as HIV-positive. [33] Performance along the treatment cascade has progressively strengthened. Of those newly testing HIV-positive in 2015–16, 80% of female sex workers and 85% of MSM were reported to have started ART. Cross-sectional data from an anonymous polling booth survey in 2017 conducted by NASCOP found 26% of FSWs to be HIV-positive, among whom 79% said they were enrolled in care and 73% on ART. For MSM, 17% were HIV-positive, 69% of whom were enrolled in care and 63% on ART [33]. Since the entry point for the HIV treatment cascade is knowledge of HIV status, the more frequent testing rates in areas with low KP:PE ratios, and more frequent outreach contacts, would be expected to result in higher coverage at subsequent steps of the cascade. Moreover, regular repeat HIV testing and STI screening provide quarterly evidence of programme effectiveness in reducing and maintaining low HIV/STI incidence.

Micro-planning empowers peer workers to systematically plan, conduct and monitor their work at the local level. [24] Methods and tools facilitate targeting, reaching and retaining KPs in prevention, care, and treatment. By building PE skills to plan and monitor, micro-planning also builds accountability and empowers KPs to take decisions. Yet, while micro-planning guidance provides a general framework, there has been limited evidence to inform guidance on operational details. Questions such as ‘how many KPs should a PE be responsible for’ and ‘how often should KPs be contacted through outreach’ are examples. This study addresses this gap by analysing routine data from a national programme implementing standardised micro-planning methods.

In Kenya, despite overall progress in scaling up the KP programme, important differences were apparent across different settings. Since the logic of micro-planning is that stronger relationships between peer educators and KPs–more frequent contacts, closer relationships, more time to promote prevention and clinic check-ups–result in better outcomes, we looked for evidence of this in the data. The results were striking, with large and significant differences seen for programmes where KP:PE ratios were less than 90 (for FSWs) and 60 (for MSM). For both FSWs and MSM, KP:PE ratios below the observed thresholds not only were associated with significantly improved outcomes such as outreach contacts, for which PEs are directly implicated, but also regular clinic attendance for a range of complementary prevention, screening, and treatment services.

Several limitations are apparent in this study of routine programme data. Quarterly data did not permit analysis of more frequent activities such as monthly outreach contacts. Limited data also impede further analysis of the weaker correlations seen between KP:PE ratios, and condom distribution and violence reporting. Our analysis of available aggregate programme data was limited to trend and stratified analyses. Additional regression analyses to identify other factors that may have influenced the outcomes, as well as cost-benefit analysis, would have been useful. However, limited number of cases, and unavailability of information on costs impeded these analyses. Despite these limitations, the effect of lower KP:PE ratios on outreach and clinical service utilisation is strong and highly significant, with important programmatic implications.

While the use of clinical services for HIV/STI testing or counselling appears to increase with more frequent promotion by PEs, uptake, and utilisation of other services may be more complicated. Condom distribution responds to demand, which is known to vary due to a range of social and economic factors. Violence reporting often increases at the start of a prevention programme [34], then drops as progress is made in reducing the incidence of violence itself. Reporting of violence by KPs increased over time but not consistently in relation to lower KP:PE ratios, perhaps because reporting of violence depends not only on peer mobilisation but also on effective violence response systems, which act simultaneously to prevent violence and improve reporting. These are important areas for future research.

## Conclusion

Kenya’s experience with implementing KP programmes supports the feasibility of rapid national scale-up, attaining high levels of coverage within a few years. Progress was facilitated by mapping and estimation exercises, which provided denominators for target setting, planning and budgeting. A standardised national approach to implementation (micro-planning), backed by a dedicated technical support unit and adequately staffed, resulted in more regular outreach contacts and higher levels of service uptake and utilisation.
